# Update on RAAS Modulation for the Treatment of Diabetic Cardiovascular Disease

**DOI:** 10.1155/2016/8917578

**Published:** 2016-08-29

**Authors:** Stella Bernardi, Andrea Michelli, Giulia Zuolo, Riccardo Candido, Bruno Fabris

**Affiliations:** ^1^Department of Medical Sciences, University of Trieste, Cattinara Teaching Hospital, Strada di Fiume, 34100 Trieste, Italy; ^2^Division of Medicina Clinica, Azienda Sanitaria Universitaria Integrata di Trieste (ASUITS), Cattinara Teaching Hospital, Strada di Fiume, 34100 Trieste, Italy; ^3^Diabetes Centre, Azienda Sanitaria Universitaria Integrata di Trieste (ASUITS), Via Puccini, 34100 Trieste, Italy

## Abstract

Since the advent of insulin, the improvements in diabetes detection and the therapies to treat hyperglycemia have reduced the mortality of acute metabolic emergencies, such that today chronic complications are the major cause of morbidity and mortality among diabetic patients. More than half of the mortality that is seen in the diabetic population can be ascribed to cardiovascular disease (CVD), which includes not only myocardial infarction due to premature atherosclerosis but also diabetic cardiomyopathy. The importance of renin-angiotensin-aldosterone system (RAAS) antagonism in the prevention of diabetic CVD has demonstrated the key role that the RAAS plays in diabetic CVD onset and development. Today, ACE inhibitors and angiotensin II receptor blockers represent the first line therapy for primary and secondary CVD prevention in patients with diabetes. Recent research has uncovered new dimensions of the RAAS and, therefore, new potential therapeutic targets against diabetic CVD. Here we describe the timeline of paradigm shifts in RAAS understanding, how diabetes modifies the RAAS, and what new parts of the RAAS pathway could be targeted in order to achieve RAAS modulation against diabetic CVD.

## 1. Introduction

Cardiovascular diseases (CVD) are the main cause of diabetes-related morbidity and mortality [1, 2]. They include myocardial infarction, which is due to premature atherosclerosis, and diabetic cardiomyopathy, both leading to heart failure. Patients with diabetes have a higher prevalence of cardiovascular morbidity and mortality as compared to the general population [3], such that diabetes is considered not only as an independent cardiovascular risk factor but also as a cardiovascular event equivalent, meaning that patients with diabetes have a risk of cardiovascular complications equal to that of patients with a prior myocardial infarction [4]. This excess cardiovascular risk in comparison to the general population is explained only partly by conventional cardiovascular risk factors, such as hyperglycemia, dyslipidemia, hypertension, and cigarette smoking.

One of the links between diabetes and such a high prevalence of CVD is renin-angiotensin-aldosterone system (RAAS) activation. It has been shown that the RAAS plays a major role in the development of diabetic cardiovascular complications [5], as it promotes atherosclerosis [6, 7], cardiomyocyte loss, and extensive myocardial fibrosis [8, 9]. Consistent with this view, ACE inhibitors and angiotensin II receptor blockers represent the first line therapy for primary and secondary CVD prevention in patients with diabetes [10]. Recent research has uncovered new dimensions of the RAAS and, therefore, new potential therapeutic targets against diabetic CVD. Here we describe the timeline of paradigm shifts in RAAS understanding, how diabetes modifies the RAAS, and what new parts of the RAAS pathway could be targeted in order to achieve RAAS modulation against diabetic CVD.

## 2. Paradigm Shifts in the Renin-Angiotensin-Aldosterone System Understanding

### 2.1. The Renin-Angiotensin-Aldosterone System Has Hemodynamic and Nonhemodynamic Actions

**Figure 1:**
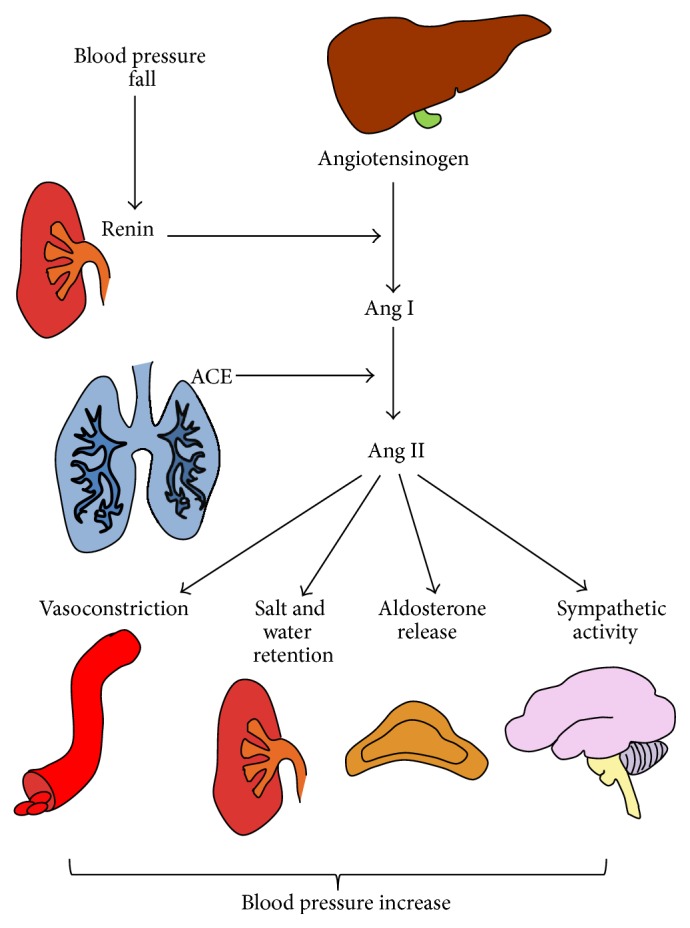
The activation of systemic renin-angiotensin-aldosterone system cascade for blood pressure control. The activation of the circulating RAAS cascade that follows a blood pressure fall begins with renin secretion by the kidney. Once it has been released into the bloodstream, renin cleaves angiotensinogen to form Ang I, which is then converted to Ang II by pulmonary ACE. Ang II stimulates vasoconstriction, renal retention of salt and water, aldosterone secretion, and sympathetic activity, whereby it increases blood pressure. ACE is for angiotensin-converting enzyme; Ang is for angiotensin; RAAS is for renin-angiotensin-aldosterone system.

The renin-angiotensin-aldosterone system (RAAS) consists of a group of enzymes and peptides whose main function is to control blood pressure by regulating vasoconstriction, sodium reabsorption, and body fluid homeostasis. The modern view of the RAAS began with the concept that this was a life-saving system, which raised blood pressure by approximately 30 mmHg in case of an acute hemorrhage [11]. Classically, the process whereby the RAAS raises blood pressure usually starts within the kidney, where a blood pressure fall stimulates renin release into the bloodstream [12, 13]. Then, circulating renin cleaves hepatic angiotensinogen and generates angiotensin (Ang) I, which is converted to Ang II by pulmonary angiotensin-converting enzyme (ACE), as represented in Figure 1 [14–16]. Soon after its generation, Ang II causes smooth muscle cell vasoconstriction, stimulates the sympathetic nervous system, and promotes renal retention of salt and water by binding to its specific receptors [17, 18]. Moreover, in the adrenal glands, Ang II stimulates the release of aldosterone, which enhances tubular sodium reabsorption in the kidney and increases the effective circulating plasma volume [19].

**Figure 2:**
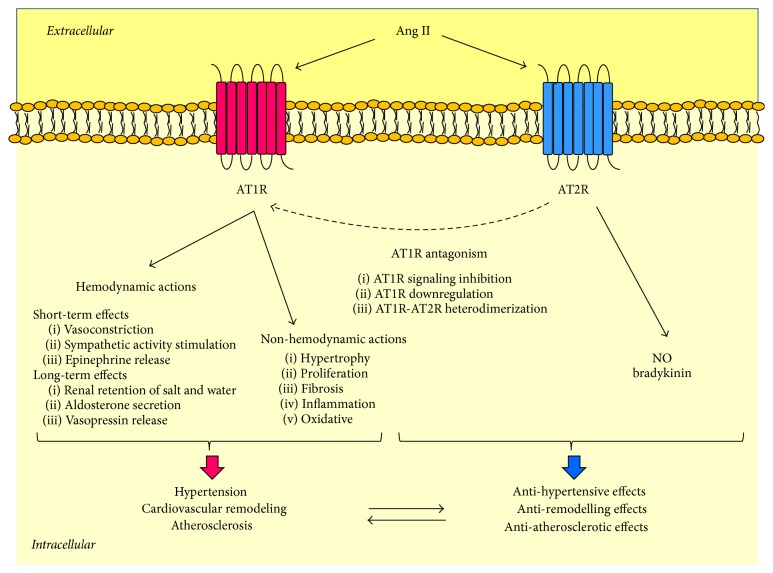
Angiotensin II receptors. Angiotensin II has two major receptor isoforms: Ang II type 1 receptor (AT1R) and Ang II type 2 receptor (AT2R). AT1R stimulation mediates the classical actions of Ang II, including hemodynamic and nonhemodynamic effects, leading to hypertension, cardiac remodeling, and atherosclerosis. On the other hand, AT2R stimulation usually causes opposing effects to AT1R. Moreover, it can antagonize AT1R by downregulating it, inhibiting its signaling, or binding to it. Ang is for angiotensin; AT1R is for Ang II type 1 receptor; AT2R is for Ang II type 2 receptor; NO is nitric oxide.

Ang II has two main receptors: Ang II type 1 receptor (AT1R) and Ang II type 2 receptor (AT2R), as represented in Figure 2. Studies in mice lacking AT1R showed that the hemodynamic actions of Ang II depended on AT1R [20–22]. On the other hand, AT2R, which was found highly expressed in differentiated mesenchymes during fetal life and decreased rapidly after birth, seemed to regulate fetal development [18, 23]. Nevertheless, AT2R is still detectable in adulthood in different organs, such as the heart, kidney, adrenal glands, brain, ovaries and uterus, and the vessels [24], where its main function is to counterbalance AT1R. For instance, AT2R-knockout mice exhibit high blood pressure levels and increased vascular sensitivity to Ang II [25, 26], suggesting that AT2R antagonizes AngII-AT1R peripheral effects. Such an action could be achieved not only by vasodilator effects that are independent of AT1R [27, 28] but also by AT1R downregulation [29] and/or direct inhibition of AT1R signaling [30, 31], as represented in Figure 2.

Although the RAAS was a life-saving system, the first aspect that became clear was that if the Ang II cascade was activated inappropriately, it could lead to hypertension and CVD [32]. Therefore, ACE inhibitors and Ang II type 1 receptor blockers (ARB) were designed as antihypertensive therapies [33, 34]. A paradigm shift in RAAS understanding occurred when the CONSENSUS and the SOLVD trials showed that the ACE inhibitor Enalapril reduced overall mortality by 27% and 16% in patients with heart failure [35, 36]. Enalapril's benefit suggested that the RAAS had significant nonhemodynamic actions that were associated with cardiovascular pathology. These trials led to mechanistic studies demonstrating that Ang II promoted ventricular hypertrophy [37–39], myocardial infarction [40], and atherosclerosis [41–43], independent of blood pressure values. In particular, by binding to AT1R, Ang II was able to induce reactive oxygen species generation, tissue inflammation and fibrosis, and the regulation of cell growth and differentiation as well as apoptosis and survival as summarized in Table 1 [41–63].

### 2.2. Circulating and Tissue Renin-Angiotensin-Aldosterone System

The observation that many tissues were capable of synthesizing the RAAS key components led to another paradigm shift in the timeline of RAAS understanding: the RAAS was not anymore only a circulating hormonal system but also a tissue system widespread in cardiovascular organs [64–68]. ACE and Ang II receptors were identified in all the relevant target tissues including the heart, kidney, blood vessels, and adrenal glands [69–71], where they have not only endocrine but also paracrine and autocrine effects [72]. Interestingly, circulating and local systems can sometimes behave in opposite ways, like in cases of high salt intake, which generally downregulates circulating and upregulates local RAAS [73].

Renin, angiotensinogen, ACE, and Ang II receptors were all present in the heart [67], where they were found upregulated in models of cardiac injury, such as volume overload [74], myocardial infarction [75], and heart failure [76–78]. For example, one of the first experimental observations was that ACE increased in heart failure, its cardiac induction was tissue specific, and it correlated with the size of myocardial infarction [76]. Interestingly, ACE was expressed not only in the cardiomyocytes adjacent to the infarct size, but also in fibroblasts, macrophages, and endothelial cells. Further studies demonstrated that the increase in cardiac ACE was functionally significant, as it was associated with an increased intracardiac conversion of Ang I to Ang II, which could potentially impair ventricular function and induce ventricular arrhythmias [79], eventually promoting heart failure.

Almost a decade after the CONSENSUS and the SOLVD studies, the HOPE [5] trial extended to high-risk patients, such as those with diabetes, the concept that RAAS blockade protected against cardiovascular morbidity and mortality. In that trial, Ramipril significantly reduced the rates of death, myocardial infarction, and stroke in patients with vascular disease or diabetes. Likewise, the LIFE trial showed that Losartan was more effective than Atenolol in reducing cardiovascular morbidity and mortality in patients with hypertension, diabetes, and ventricular hypertrophy [80]. Also the EUROPA [81] and ADVANCE [82] trials provided evidence that an ACE inhibitor treatment improved survival and reduced the risk of major cardiovascular events in patients with diabetes. Consistent with these results, experimental studies demonstrated that Ang II blockade significantly reduced cardiac damage [8, 9] and atherosclerotic plaque accumulation [6, 7] in the context of diabetes. More recently, the ONTARGET trial has evaluated whether the combination of an ACE inhibitor with an ARB was better than the full dose of either drug. In the end, there was no superiority of the ACE inhibitor versus the ARB in terms of cardiovascular events. Moreover, dual blockade did not confer greater cardiovascular protection and it actually put risk of more adverse events [83].

At the same time the RALES and EPHESUS trials showed that aldosterone antagonism on top of Ang II blockade provided a major additive benefit as it reduced significantly overall mortality and the rate of death from cardiovascular causes among patients with severe heart failure [84] or after acute myocardial infarction [85]. These results suggested that Ang II blockade could not totally suppress the production of aldosterone and that other factors in addition to Ang II were important in the production of this hormone. Moreover, they reinforced the notion that aldosterone promoted organ damage by a series of cellular and tissue effects, elicited by binding predominantly to the mineralocorticoid receptors, which are reported in Table 1 [86–103]. More recently, Eplerenone benefits have been extended to diabetic patients, where this treatment reduced adverse CV events in diabetic patients with heart failure following myocardial infarction [104]. A randomized study evaluating the effects of two aldosterone antagonists, Finerenone versus Eplerenone, in diabetic patients with heart failure is still ongoing [105].

### 2.3. ACE2 and (Pro)Renin Discovery

**Figure 3:**
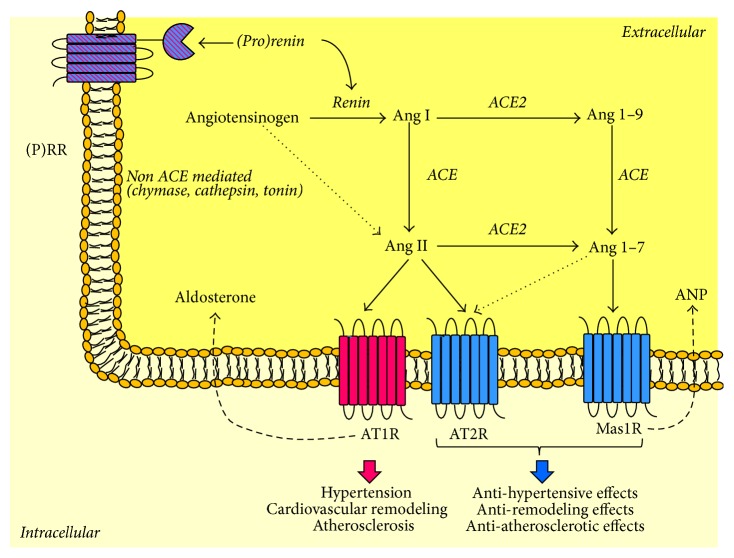
Tissue renin-angiotensin-aldosterone system. The activation of the RAAS cascade begins with renin secretion. Renin is secreted as a precursor protein, prorenin. Renin and prorenin can bind to their specific receptor called (P)RR and activate intracellular pathways independent of RAAS. Otherwise, renin cleaves angiotensinogen to form Ang I, which is then converted to Ang II by ACE. Ang II binds to its specific receptors: AT1R and AT2R. AT1R promotes blood pressure increase, cardiac remodeling, and atherosclerosis development. In addition, through AT1R, Ang II stimulates aldosterone secretion. On the other hand, AT2R seems to antagonize these effects. Moreover, Ang I and Ang II can be cleaved by ACE2 to form Ang 1–9 and Ang 1–7, which have opposite effects to those of Ang II, such as vasodilation, anti-inflammatory, antifibrotic, and antiremodeling effects, which are mediated by Mas1R and partly by AT2R. In addition, Ang 1–7 stimulates local ANP secretion. ACE is for angiotensin-converting enzyme; ACE2 is for angiotensin-converting enzyme 2; Ang is for angiotensin; AT1R is for Ang II type 1 receptor; AT2R is for Ang II type 2 receptor; ANP is for atrial natriuretic peptide; (P)RR is for (pro)renin receptor.

Nevertheless, ACE2 discovery represents perhaps the last fundamental paradigm shift in RAAS understanding [106, 107]. ACE2 is a carboxypeptidase whose main function is to degrade Ang II to generate Ang 1–7, as represented in Figure 3. Although ACE2 can also degrade Ang I to generate Ang 1–9, its catalytic efficiency is 400-fold higher with Ang II, and therefore its main effect is the degradation of Ang II to Ang 1–7 [108, 109]. Ang 1–7 is a biologically active peptide, which exerts opposite peripheral actions to those of Ang II [110] by binding predominantly to the Mas1 receptor (Mas1R) [111]. Cellular and tissue effects of Ang 1–7/Mas1R are summarized in Table 1 [112–128]. Moreover, Ang 1–7, like all Ang II “breakdown” products, has the potential to act as an endogenous ligand of AT2R [129], further promoting organ protection (Figure 3).

The identification of ACE2 provided evidence that the RAAS had two pathways with opposite effects: the classic ACE/Ang II/AT1R and the new ACE2/Ang 1–7/Mas1R (and AT2R) pathway. Accordingly, the current scientific opinion is that what is critical in CVD development is an imbalance in ACE-Ang II and ACE2-Ang 1–7 [130]. Consistent with this view, ACE2 is regarded as the central regulator of the RAAS [131], as its changes can affect not only Ang II detrimental actions but also Ang 1–7 protective effects. For instance, a decrease in ACE2 results in activation of the Ang II/AT1R axis with a parallel reduction of Ang 1–7 [51, 132]. Nevertheless, ACE2 protective effects can be attributed not only to the degradation of Ang II and the generation of Ang 1–7 or to the degradation of Ang I and the generation of Ang 1–9 [106], as represented in Figure 3, but also to the local increase of ANP. Recently, we have shown that ACE2 regulates ANP production through Ang 1–7 [132].

Having said that, the final entry in the RAAS is the (pro)renin receptor [(P)RR], which is a specific receptor for both renin and its inactive precursor prorenin [133], collectively called (pro)renin (Figure 3). When (pro)renin binds to (P)RR it can degrade angiotensinogen to Ang I, but it can also trigger intracellular signaling pathways, which are independent of Ang II generation [133]. Interestingly, the ability of prorenin to activate the RAAS depends on the fact that its binding induces a conformational change that exposes prorenin active site, which becomes catalytically active and generates Ang I. The biology of (P)RR is very complex and only partially understood [134]. The association between (P)RR gene polymorphism and ambulatory BP in Japanese men [135] has suggested that (P)RR was related to cardiovascular disease leading to experimental studies supporting this hypothesis. The cellular and tissue effects of (pro)renin are reported in Table 1 [136–145]. Nevertheless, transgenic animals overexpressing (pro)renin developed hypertension that was sensitive to ACE inhibition, indicating that this effect was due to Ang II generation, which led to arguing against an independent role of (P)RR in CVD [146, 147]. On the other hand, Batenburg and Danser have suggested that (pro)renin overexpression led to a 400-fold increase of plasma (pro)renin levels, which is far from the 1000-fold–100.000-fold increase that is required to observe (pro)renin effects* in vitro* [148]. Certainly, (P)RR has essential functions that are independent of the RAAS and are related to the vacuolar H^+^-proton adenosine triphosphatase (V-ATPase) [149]. In particular, (P)RR is a cofactor of the V-ATPase [150] and is required for central nervous system development [151, 152]. (P)RR is also an essential partner in Wnt receptor complex signaling [153], which regulates normal pattering of the embryo, and in adults, cell proliferation, migration, polarity, and tissue repair. These findings explain not only why (P)RR deletion yielded embryos that died before the end of embryogenesis [153, 154] but also why mice with specific ATP6ap2/(P)RR knockout in cardiomyocytes died of severe heart failure within 3 weeks of age [155].

## 3. Diabetes, the Activation and Imbalance of the Renin-Angiotensin-Aldosterone System, and Cardiovascular Disease

### 3.1. The Renin-Angiotensin-Aldosterone System Activation Links Diabetes to CVD

The current picture of the RAAS is that of an extremely complex pathway whose overstimulation triggers a chain of events contributing to cardiovascular disease. The RAAS is found not only in the circulation, where it has hemodynamic effects, but also at a tissue level, where it has nonhemodynamic effects. It is activated in the hypertrophied, ischemic, and failed heart, and activation of the ACE/AngII/AT1R axis leads to both accelerated atherosclerosis and direct cardiac injury, as shown by infusing Ang II or by the studies where RAAS blockade was able to attenuate or prevent cardiac damage [78].

Patients with diabetes have a higher prevalence of cardiovascular morbidity and mortality as compared to the general population [3]. On one hand, diabetes is associated with accelerated atherosclerosis affecting the coronaries, which increases the risk for myocardial infarction and heart failure. On the other hand, it can cause diabetic cardiomyopathy, which corresponds to a cardiac dysfunction independent of coronary artery disease, hypertension, and valvular complications [156].

Upregulation of the RAAS is evident in diabetes. Miller and colleagues demonstrated that during the early stages of diabetes there was an increase in plasma renin activity, mean arterial pressure, and renal vascular resistance [157]. Moreover, the fact that Losartan lowered blood pressure more in hyperglycemic than in euglycemic conditions [158], and that Captopril and Eprosartan caused a greater renal vasodilator response during hyperglycemia [159], suggested that glucose levels were associated with an activation of the RAAS, which made diabetic patients more sensitive to RAAS antagonism. Consistent with this view, direct renin inhibition with Aliskiren led to significant improvement of left ventricular hypertrophy [160] and end-systolic volume [161] only in groups of patients with diabetes.

The importance of RAAS antagonism in the prevention/reduction of cardiovascular complications has clearly demonstrated that diabetes-induced RAAS activation contributes substantially to diabetic CVD [5, 80–82]. Activation of AngII/AT1R pathway in the setting of diabetes can in fact promote cell growth and proliferation, apoptosis [162], oxidative stress generation [9], inflammation [6], and fibrosis [9], which are all leading to cardiac remodeling and atherosclerosis, that can be reversed/reduced by RAAS blockade [7–9].

### 3.2. Diabetes-Induced Activation of the AngII/AT1R Pathway and Imbalance of the Renin-Angiotensin-Aldosterone System

There are several mechanisms whereby diabetes can promote tissue Ang II/AT1R actions. Firstly, hyperglycemia directly stimulates local Ang II production in cardiomyocytes [163], cardiac fibroblasts [164], and endothelial cells [165] as well as in murine and human diabetic heart tissues [164, 166]. For example, Fiordaliso and colleagues demonstrated that glucose increased Ang II in cardiac myocytes, which made them more susceptible to undergo apoptosis [167], consistent with the view that the RAAS promotes the development of diabetic cardiomyopathy [162, 166]. Studies on cardiac myocytes suggest that the mechanism whereby hyperglycemia increases local Ang II in the heart is the generation of intracellular Ang II by intracellular chymase and/or internalized prorenin. Then, intracellular Ang II could directly produce oxidative stress and cellular apoptosis and/or enhance RAAS components expression through a positive feedback mechanism [168].

The second mechanism underlying AngII/AT1R activation in diabetes is that high glucose concentrations can enhance the tissue response to Ang II and vice versa. While Ang II can increase the activation of glucose-induced transcription factors in vascular smooth muscle cells [169], hyperglycemia can increase the contractile aortic response to Ang II [170]. This additive effect could be ascribed to the fact that diabetes induces AT1R expression in the heart [171, 172] and the vasculature [172]. Interestingly, several works have demonstrated that hyperglycemia can also stimulate aldosterone secretion by increasing local Ang II. Xue and Siragy observed an upregulation of renal aldosterone synthase in diabetic rats, which was significantly reduced by AT1R blockade [173]. Similar findings were reported in podocytes [174] and in cardiomyocytes [175]. Locally, aldosterone triggers a vicious cycle that promotes AngII/AT1R effects as it mediates part of Ang II effects [176], increases tissue ACE and AT1R [177], and reduces tissue ACE2 [178, 179]. Moreover, together with Ang II, aldosterone promotes oxidative stress, fibrosis, and apoptosis. Therefore, many studies are now supporting at least a synergistic role for aldosterone in the pathogenesis of diabetic cardiovascular complications [180].

The third way whereby diabetes promotes Ang II tissue actions is through the several metabolic abnormalities associated with hyperglycemia. These include advanced glycation end products, which form after prolonged hyperglycemia and oxidative stress, dyslipidemia, and low-grade inflammation. All of them can in fact stimulate the AngII/AT1R pathway by upregulating AT1R expression [181–184]. An interesting aspect that should be considered is that, in the heart, the conversion of Ang I to Ang II relies not only on ACE activity but also on other enzymes such as tissue chymase [185, 186], which are found primarily in mast cells. Given this localization, chymase might link inflammation to RAAS activation and CVD. Unfortunately, inhibition of this chymase is yet to be realized [187].

Another intriguing mechanism whereby diabetes enhances AngII/AT1R actions is ACE2 downregulation, which does not only promote Ang II actions but also reduce local Ang 1–7 leading to an imbalance of the RAAS. This concept is supported by the works of Tikellis and colleagues, who showed that the induction of diabetes was associated with a significant reduction of ACE2 expression and ACE2 activity in the heart and the vasculature [172] together with a significant increase in circulating Ang II and a significant reduction of Ang 1–7 levels [172]. Similar changes were reported in the kidney of diabetic mice [132, 188]. It has been argued that ACE2 may be more important than ACE in regulating cardiac levels of Ang II and Ang 1–7 and therefore more important for balancing RAAS activation [189]. This concept relies on the experimental evidence that while ACE2 deficiency results in increased cardiac levels of Ang II [51] and reduced levels of Ang 1–7 [132], ACE deficiency does not modify cardiac Ang II, which could be generated by non-ACE pathways [190], including chymases.

Therefore, ACE2 replenishing strategies could be an important therapeutic tool against diabetic CVD. Another promising therapeutic strategy against diabetic CVD seems to be AT2R stimulation. The rationale behind it is that diabetes seems to upregulate AT2R expression [24]. AT2R expression was found increased in the hearts of diabetic rats [171, 191], as well as in their kidneys [192]. Similar results were obtained in kidney biopsies of patients with type 2 diabetes [193]; this is in line with the report that glucose induced AT2R renal expression both* in vivo* [194, 195] and* in vitro* through the expression of the transcriptor factor IRF-1 [195].

Lastly, diabetic patients have higher prorenin levels than normal subjects, and their prorenin levels are 10-fold higher than their renin levels [196]. Given that the binding of prorenin to (P)RR can generate Ang I and also stimulate (P)RR intracellular signaling, it has been argued that plasma prorenin could contribute more substantially than renin to the pathogenesis of end-organ damage [196]. This is supported by the finding that diabetic patients with high prorenin levels had a higher risk of microvascular complications [197], whose development could be predicted by the same prorenin levels [198]. Consistent with these circulating changes, Connelly and colleagues demonstrated that, also at a tissue level, diabetic cardiomyopathy was associated with a 3-fold increase in both (P)RR gene and protein expression, which were reduced by the renin inhibitor Aliskiren. Although the increased expression of (P)RR in the diabetic heart was interpreted as a beneficial response to limit intracellular acidosis, due to its sequence identity with the V-ATPase, it was argued that (P)RR abundance could still modulate the activity of the local RAAS and expose cardiac myocytes to Ang II increased concentrations [199].

## 4. New Potential Therapies against Diabetic Cardiovascular Complications

### 4.1. New Agents for Classic Targets

#### 4.1.1. Angiotensin Receptor-Neprilysin Inhibition

**Figure 4:**
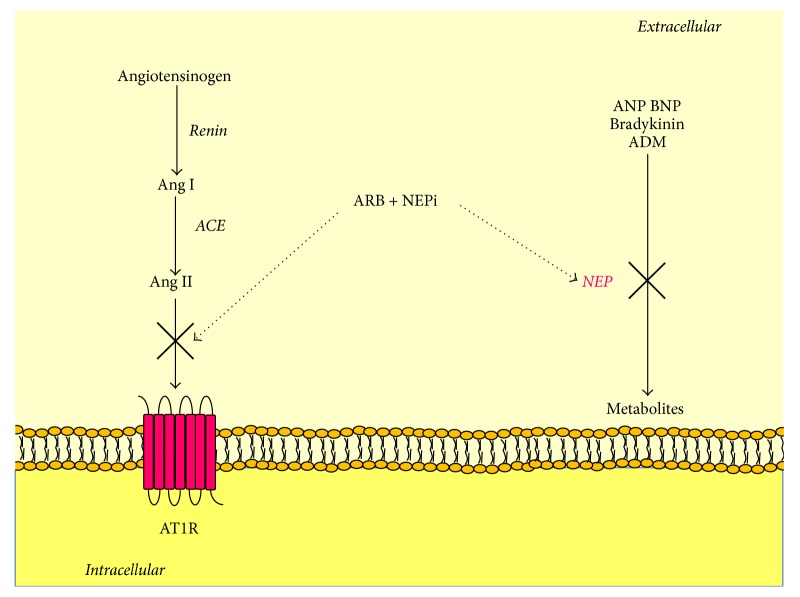
Mechanisms of action of angiotensin receptor/neprilysin inhibitors. ARB antagonize Ang II binding to AT1R, which causes hypertension and multiorgan injury. NEPi inhibit the activity of NEP, which is an enzyme degrading natriuretic peptide, bradykinin, and other peptides. The result is a reduction of Ang II harmful effects and an increase of the benefits of natriuretic peptides and bradykinin. ACE is for angiotensin-converting enzyme; ADM is for adrenomedullin; Ang is for angiotensin; ANP is for atrial natriuretic peptide; ARB is for angiotensin II receptor blockers; AT1R is for Ang II type 1 receptor; BNP is for brain natriuretic peptide; NEPi is for neprilysin inhibitors.

ACE inhibition, AT1R antagonism, and MR blockade are some of the most classic therapeutic strategies against CVD. Another classic therapeutic strategy against heart failure is to increase natriuretic peptide levels, as they are natriuretic, diuretic, vasodilating, and able to inhibit pathologic growth in heart failure [200]. However, although the idea to boost their activity in patients with heart failure seemed theoretically infallible, the approaches used for years have produced only partial if not disappointing effects. These approaches included short-term intravenous infusions of natriuretic peptides or inhibition of neprilysin, which is the enzyme that degrades natriuretic peptides, together with bradykinin, adrenomedullin, and possibly other substrates such as Ang II. So, the most likely explanation underlying the disappointing effect of the oral inhibitor of neprilysin was that neprilysin degraded also Ang II, and therefore inhibiting neprilysin would increase not only natriuretic peptides but also Ang II actions. Eventually, the combination of an ACE and a neprilysin inhibitor solved this problem and turned out to be superior to either approach in terms of cardiovascular benefits [201, 202]. Unfortunately, in clinical trials this combination was associated with angioedema [203, 204]. In order to overcome this issue, angiotensin receptor-neprilysin inhibitors (ARNis) have been developed, such as LCZ696, which is an association of the ARB Valsartan with the neprilysin inhibitor Sacubitril (Figure 4). In a recent trial this association was found superior to Enalapril in reducing the risk of death and hospitalization in patients with heart failure [205], including those with diabetes [206]. This is consistent with the observation that the administration of LCZ696 in an experimental model of diabetes significantly reduced cardiac hypertrophy and fibrosis and improved ejection fraction after myocardial reperfusion injury [207].

#### 4.1.2. Aldosterone Synthase Inhibition

Another classic target against CVD is aldosterone, due to the growing appreciation of its contribution to CVD development and progression. The drugs that are available for blocking aldosterone actions are Spironolactone and Eplerenone, which are both mineralocorticoid-receptor antagonists. Nevertheless, Spironolactone and Eplerenone have still limited clinical use, due to the poor selectivity of Spironolactone, the low potency of Eplerenone, and the fact that only MR-dependent effects of aldosterone can be inhibited. An alternative approach to antagonize aldosterone is inhibiting its formation. For this reason, several aldosterone synthase (CYP11B2) inhibitors have been developed. The compounds targeting CYP11B2 that have been studied so far are FAD286, which has been used mostly in experimental settings, and LCI699, which has been developed for human use in 2010 [208]. A few works have demonstrated that FAD286 reduced mortality, improved cardiac remodeling [209, 210], and reduced atherosclerosis [211]. Nevertheless, both drugs have exhibited clear side effects due to the inhibition of CYP11B1, which synthesizes glucocorticoids. Moreover, another concern prior to their use is that aldosterone synthase inhibitors could allow the activation of unprotected MR by glucocorticoids, and this needs further research.

#### 4.1.3. Renin Inhibition

Renin inhibition represents another very attractive therapeutic strategy to reduce Ang II levels. This can be achieved with the renin inhibitor Aliskiren, which attenuates Ang II generation and reduces blood pressure comparably to ACE inhibitors and ARB [212]. However, similarl to what was reported in the ONTARGET trial, the ALTITUDE study has demonstrated that the simultaneous administration of Aliskiren with an ACE inhibitor or an ARB should be avoided [213]. A critical concern on the use of Aliskiren is that it is associated with high renin levels that might bind to (P)RR [214, 215]. Therefore, specific blockade of (P)RR could provide more benefits as compared to renin inhibition alone, as it could not only reduce (pro)renin enzymatic activity but also prevent some AngII-independent effects of (pro)renin. So far, a (P)RR antagonist, called HRP (“handle region” peptide), has been designed. In particular, this molecule mimics the “handle region” of prorenin, which allows its binding to (P)RR and its subsequent nonproteolytic activation. Studies in animal models of diabetes show promising results as reviewed in [148], possibly because HRP exerts its effects only in diseases associated with high prorenin and low renin levels, such as diabetes.

### 4.2. New Agents for New Targets

#### 4.2.1. ACE2 Replenishing Strategies

A promising therapeutic strategy in cardiovascular medicine is represented by RAAS modulation. As compared to RAAS antagonism, RAAS modulation combines ACE/AngII/AT1R blockade with the stimulation of ACE2/Ang 1-7/Mas1R and AT2R. The latter can be achieved by a series of new therapies that includes ACE2 replenishing strategies, Ang 1–7 administration, and AT2R agonists. However, also ACE inhibitors and AT1R blockers should be considered RAAS modulating agents, as they can increase ACE2 expression and Ang 1–7 levels severalfold, suggesting that part of their beneficial effects is due to ACE2/Ang 1–7 effects [216, 217].

Having said that, Crakower and colleagues showed that ACE2 is an essential regulator of cardiac function, by demonstrating that ACE2 deficiency was associated with reduced systolic function [218]. Similar findings have been reported in animal models of type 1 and type 2 diabetes [219, 220]. In addition to that, we have demonstrated that ACE2 deficiency was associated with accelerated atherosclerosis [51]. It has also to be noted that ACE2 deficiency might in fact influence not only the risk of developing CVD but also the response to the treatment, particularly in the setting of diabetes where ACE2 expression is constitutively low [188]. These findings emphasize the potential utility of ACE2 repletion as a strategy to reduce CVD. Current therapeutic tools that modulate ACE2 levels or activity, which are still under investigation, include adenoviral ACE2 gene transfer, recombinant human ACE2 (rhACE2), ACE2 activators, oral ACE2, and Ang 1–7 bioencapsulated in plant cells. So far, both ACE2 gene transfer and the administration of an ACE2 activator have ameliorated diabetic cardiomyopathy [221, 222]. Moreover, the reports that rhACE2 administered intravenously to healthy human subjects was well tolerated [223] and that it resulted in sustained reduction in plasma Ang II levels and elevation in Ang 1–7 levels [131] are encouraging, as they are shortening the gap between bench and bedside with respect to the possibility of using rhACE2 in clinical practice.

#### 4.2.2. Ang 1–7 Administration

Based on the protective cardiovascular effects of Ang 1–7, several experimental studies have tested the hypothesis that Ang 1–7 infusion could ameliorate diabetic cardiomyopathy. In all these works, Ang 1–7 improved all the structural hallmarks of diabetic cardiomyopathy, which is characterized by left ventricle hypertrophy and left and right ventricle fibrosis and dysfunction [224–226]. In db/db mice with early [227] and advanced disease [228], Ang 1–7 reversed diabetes-induced changes. In particular, in the early disease model [227], Ang 1–7 increased cardiac output and cardiac index and decreased cardiomyocyte hypertrophy, heart fibrosis, and inflammation. Vascularization was also improved, which correlated with increased numbers of bone marrow residing in and circulating endothelial and mesenchymal stem cells [227]. Here, Mas1R seemed to be the main receptor mediating Ang 1–7 effects, which explains why AVE0991, which is a Mas1R agonist, had cardioprotective effects in diabetic rats [229]. In addition, in the advanced disease model, Ang 1–7 ameliorated diastolic function and decreased not only cardiac hypertrophy and fibrosis but also triacylglycerol accumulation [228]. Moreover, consistent with the report that Ang 1–7 preserved cardiac function, coronary perfusion, and aortic endothelial function in a rat model of heart failure [230], Ang 1–7 improved cardiac recovery from ischemia/reperfusion and restored the normal reactivity to constrictor and dilator stimuli in the vasculature [231]. Recently, we have shown that Ang 1–7 administration significantly reduced* ex vivo* leukocyte recruitment in an animal model of diabetes. This was associated with a reduction of glucose-induced molecule adhesion expression and leukocyte adhesion to endothelial cells* in vitro* [232]. These effects were completely blocked by the Mas1R antagonist, suggesting that Mas1R was the main receptor mediating Ang 1–7 effects on endothelial cells.

#### 4.2.3. AT2R Agonists

AT2R activation has been currently achieved by compound 21 (C21), which is a nonapeptide that acts as a highly selective AT2R agonist and stimulates AT2 receptors. Several studies have shown its efficacy in reducing tissue fibrosis in the cardiovascular system. However, those that have been carried out in diabetic conditions have explored its actions mostly against renal fibrosis. In this setting, C21 was able to significantly reduce renal fibrosis in experimental models of both type 1 [233] and type 2 diabetes [234]. In one of these studies, C21 was able to significantly reduce also the expression of several inflammatory mediators [233]. This finding is consistent with the vascular effects of this drug, which prevented endothelial inflammation and reduced leukocyte adhesion both* in vivo* and* in vitro* [235]. In addition, recently, C21 was found to be able to reduce diabetes-associated atherosclerosis [236].

## 5. Conclusions

The RAAS has been studied for more than a century. Nevertheless, the current picture of the RAAS is that of an extremely complex pathway, which has not been fully characterized yet and might hold in store new aspects that have still to be discovered. Certainly, what we do know is that blocking Ang II reduces cardiovascular complications. This is particularly true in diabetic conditions, where Ang II/AT1R pathway is activated, while the Ang 1–7/Mas1R is not. Therefore, the aim of new therapies is not only to block Ang II harmful effects but also to augment the activity and actions of potentially beneficial pathways, by ACE2 replenishing strategies, Ang 1–7 administration, and AT2R agonists. Here is another paradigm shift: treatment of diabetic cardiovascular disease should move from RAAS inhibition to RAAS modulation.

## Figures and Tables

**Table 1:** Cellular and tissue effects of Ang II, Ang 1–7, aldosterone, and (pro)renin in normal conditions.

	Ang II (via AT1R^*∗*^)	Ang 1–7 (via Mas1R^*∗*^)	Aldosterone (via MR^*∗*^)	(Pro)renin (via (P)RR^*∗*^)
Cardiomyocytes	Hypertrophy [44]	Hypertrophy inhibition [112, 113]	Hypertrophy [86] Apoptosis [87] Oxidative stress [87]	Hypertrophy [136, 137] Hyperplasia [138] Cell elongation [139]

Cardiac fibroblasts	Proliferation [44] Extracellular matrix production [45–48]	Antiproliferative effects [114, 115] Inhibition of collagen production [115]	Proliferation and migration [88] Extracellular matrix production (collagen/elastin) [48, 89, 90]	

Endothelial cells	Oxidative stress [49] Inflammation [50, 51]	Nitric oxide production [116] Anti-inflammatory effects [117]	Oxidative stress [91] Inflammation [92]	Hyperplasia [140] Survival [140]

Smooth muscle cells	Oxidative stress [52] Hypertrophy [53, 54] Proliferation [55] Migration [56, 57] Extracellular matrix production [58]	Antiproliferative effects [118, 119]	Proliferation [93] Migration [94] Extracellular matrix production [95, 96]	Hyperplasia [141, 142] Survival [142] Oxidative stress [142]

Macrophages	Inflammation [51, 59]	Anti-inflammatory effects [120]	Inflammation [97] Oxidative stress [98]	Inflammation [143]

Heart	Hypertrophy [60] Fibrosis [61] Apoptosis [62, 63]	Antiarrhythmic effects [121] Antifibrotic effects [122] Antiremodeling effects [123–126]	Hypertrophy [99] Fibrosis [100] Proarrhythmogenic [101] Inflammation [102]	Cardiac function deterioration [144] Fibrosis [144] Angiogenesis [144]

Vessels	Impaired vascular relaxation to Ach [52] Atherosclerosis [41–43]	Vasodilation [127, 128] Antiatherosclerotic effects [128]	Impaired vascular relaxation to Ach [91] Atherosclerosis [103]	Angiogenesis [140, 145]

^*∗*^Cellular and tissue effects are predominantly mediated by the indicated receptors.

Ang is for angiotensin; AT1R is for Ang II type 1 receptor; Mas1R is for Mas1 receptor; MR is for mineralocorticoid receptor; and (P)RR is for (pro)renin receptor.

## References

[1] Garcia M. J., McNamara P. M., Gordon T., Kannell W. B. (1974). Morbidity and mortality in diabetics in the Framingham population. Sixteen year follow up study. *Diabetes*.

[2] Russell N. D. F., Cooper M. E. (2015). 50 years forward: mechanisms of hyperglycaemia-driven diabetic complications. *Diabetologia*.

[3] Grundy S. M., Benjamin I. J., Burke G. L. (1999). Diabetes and cardiovascular disease: a statement for healthcare professionals from the american heart association. *Circulation*.

[4] Schramm T. K., Gislason G. H., Køber L. (2008). Diabetes patients requiring glucose-lowering therapy and nondiabetics with a prior myocardial infarction carry the same cardiovascular risk: a population study of 3.3 million people. *Circulation*.

[5] Yusuf S., Sleight P., Pogue J., Bosch J., Davies R., Dagenais G. (2000). Effects of an angiotensin-converting-enzyme inhibitor, ramipril, on cardiovascular events in high-risk patients. The Heart Outcomes Prevention Evaluation Study Investigators. *The New England Journal of Medicine*.

[6] Candido R., Jandeleit-Dahm K. A., Cao Z. (2002). Prevention of accelerated atherosclerosis by angiotensin-converting enzyme inhibition in diabetic apolipoprotein E-deficient mice. *Circulation*.

[7] Candido R., Allen T. J., Lassila M. (2004). Irbesartan but not amlodipine suppresses diabetes-associated atherosclerosis. *Circulation*.

[8] Matsusaka H., Kinugawa S., Ide T. (2006). Angiotensin II type 1 receptor blocker attenuates exacerbated left ventricular remodeling and failure in diabetes-associated myocardial infarction. *Journal of Cardiovascular Pharmacology*.

[9] Fiordaliso F., Cuccovillo I., Bianchi R. (2006). Cardiovascular oxidative stress is reduced by an ACE inhibitor in a rat model of streptozotocin-induced diabetes. *Life Sciences*.

[10] Mancia G., Fagard R., Narkiewicz K. (2013). 2013 ESH/ESC guidelines for the management of arterial hypertension: the Task Force for the management of arterial hypertension of the European Society of Hypertension (ESH) and of the European Society of Cardiology (ESC). *European Heart Journal*.

[11] Helwig J., Rhoads J. E., Roberts B. (1956). The metabolic response to trauma. *Annual Review of Medicine*.

[12] Friis U. G., Jensen B. L., Aas J. K., Skøtt O. (1999). Direct demonstration of exocytosis and endocytosis in single mouse juxtaglomerular cells. *Circulation Research*.

[13] Friis U. G., Jensen B. L., Sethi S., Andreasen D., Hansen P. B., Skøtt O. (2002). Control of renin secretion from rat juxtaglomerular cells by cAMP-specific phosphodiesterases. *Circulation Research*.

[14] Braun-Menendez E., Fasciolo J. C., Leloir L. F., Muñoz J. M. (1940). The substance causing renal hypertension. *The Journal of Physiology*.

[15] Braun-Menendez E., Page I. H. (1958). Suggested revision of nomenclature—angiotensin. *Science*.

[16] Page I. H., Helmer O. M. (1940). A crystalline pressor substance (Angiotonin) resulting from the reaction between renin and renin-activator. *Journal of Experimental Medicine*.

[17] Whitebread S., Mele M., Kamber B., de Gasparo M. (1989). Preliminary biochemical characterization of two angiotensin II receptor subtypes. *Biochemical and Biophysical Research Communications*.

[18] de Gasparo M., Catt K. J., Inagami T., Wright J. W., Unger T. (2000). International union of pharmacology. XXIII. The angiotensin II receptors. *Pharmacological Reviews*.

[19] Williams G. H. (2005). Aldosterone biosynthesis, regulation, and classical mechanism of action. *Heart Failure Reviews*.

[20] Ito M., Oliverio M. I., Mannon P. J. (1995). Regulation of blood pressure by the type 1A angiotensin II receptor gene. *Proceedings of the National Academy of Sciences of the United States of America*.

[21] liverio M. I., Kim H.-S., Ito M. (1998). Reduced growth, abnormal kidney structure, and type 2 (AT2) angiotensin receptor-mediated blood pressure regulation in mice lacking both AT_1A_ and AT_1B_ receptors for angiotensin II. *Proceedings of the National Academy of Sciences of the United States of America*.

[22] Oliverio M. I., Madsen K., Best C. F. (1998). Renal growth and development in mice lacking AT(1A) receptors for angiotensin II. *American Journal of Physiology-Renal Physiology*.

[23] Shanmugam S., Corvol P., Gasc J.-M. (1996). Angiotensin II type 2 receptor mRNA expression in the developing cardiopulmonary system of the rat. *Hypertension*.

[24] Jones E. S., Vinh A., McCarthy C. A., Gaspari T. A., Widdop R. E. (2008). AT2 receptors: functional relevance in cardiovascular disease. *Pharmacology and Therapeutics*.

[25] Hein L., Barsh G. S., Pratt R. E., Dzau V. J., Kobilka B. K. (1995). Behavioural and cardiovascular effects of disrupting the angiotensin II type-2 receptor gene in mice. *Nature*.

[26] Ichiki T., Labosky P. A., Shiota C. (1995). Effects on blood pressure and exploratory behaviour of mice lacking angiotensin II type-2 receptor. *Nature*.

[27] Tsutsumi Y., Matsubara H., Masaki H. (1999). Angiotensin II type 2 receptor overexpression activates the vascular kinin system and causes vasodilation. *Journal of Clinical Investigation*.

[28] Siragy H. M., Inagami T., Ichiki T., Carey R. M. (1999). Sustained hypersensitivity to angiotensin II and its mechanism in mice lacking the subtype-2 (AT2) angiotensin receptor. *Proceedings of the National Academy of Sciences of the United States of America*.

[29] Tanaka M., Tsuchida S., Imai T. (1999). Vascular response to angiotensin II is exaggerated through an upregulation of AT1 receptor in AT2 knockout mice. *Biochemical and Biophysical Research Communications*.

[30] Bedecs K., Elbaz N., Sutren M. (1997). Angiotensin II type 2 receptors mediate inhibition of mitogen-activated protein kinase cascade and functional activation of SHP-1 tyrosine phosphatase. *Biochemical Journal*.

[31] AbdAlla S., Lother H., Abdel-tawab A. M., Quitterer U. (2001). The angiotensin II AT2 receptor is an AT1 receptor antagonist. *Journal of Biological Chemistry*.

[32] Seravalle G., Cattaneo B. M., Giannattasio C. (1993). RAA system and cardiovascular control in normal subjects, hypertensives and patients with congestive heart failure. *Journal of Human Hypertension*.

[33] Ondetti M. A., Rubin B., Cushman D. W. (1977). Design of specific inhibitors of angiotensin converting enzyme: new class of orally active antihypertensive agents. *Science*.

[34] Timmermans P. B. M. W. M., Duncia J. V., Carini D. J. (1995). Discovery of losartan, the first angiotensin II receptor antagonist. *Journal of Human Hypertension*.

[35] The CONSENSUS Trial Study Group (1987). Effects of enalapril on mortality in severe congestive heart failure. Results of the Cooperative North Scandinavian Enalapril Survival Study (CONSENSUS). *The New England Journal of Medicine*.

[36] The SOLVD Investigators (1991). Effect of enalapril on survival in patients with reduced left ventricular ejection fractions and congestive heart failure. *The New England Journal of Medicine*.

[37] Ikeda Y., Nakamura T., Takano H. (2000). Angiotensin II-induced cardiomyocyte hypertrophy and cardiac fibrosis in stroke-prone spontaneously hypertensive rats. *Journal of Laboratory and Clinical Medicine*.

[38] Yamazaki T., Yazaki Y. (1999). Role of tissue angiotensin II in myocardial remodelling induced by mechanical stress. *Journal of Human Hypertension*.

[39] Mazzolai L., Pedrazzini T., Nicoud F., Gabbiani G., Brunner H.-R., Nussberger J. (2000). Increased cardiac angiotensin II levels induce right and left ventricular hypertrophy in normotensive mice. *Hypertension*.

[40] Gavras H., Lever A. F., Brown J. J., Macadam R. F., Robertson J. I. S. (1971). Acute renal failure, tubular necrosis, and myocardial infarction induced in the rabbit by intravenous angiotensin II. *The Lancet*.

[41] Daugherty A., Cassis L. (1999). Chronic angiotensin II infusion promotes atherogenesis in low density lipoprotein receptor -/- mice. *Annals of the New York Academy of Sciences*.

[42] Daugherty A., Manning M. W., Cassis L. A. (2000). Angiotensin II promotes atherosclerotic lesions and aneurysms in apolipoprotein E-deficient mice. *Journal of Clinical Investigation*.

[43] Weiss D., Kools J. J., Taylor W. R. (2001). Angiotensin II-induced hypertension accelerates the development of atherosclerosis in ApoE-deficient mice. *Circulation*.

[44] Sadoshima J.-I., Izumo S. (1993). Molecular characterization of angiotensin II-induced hypertrophy of cardiac myocytes and hyperplasia of cardiac fibroblasts critical role of the AT1 receptor subtype. *Circulation Research*.

[45] Villarreal F. J., Kim N. N., Ungab G. D., Printz M. P., Dillmann W. H. (1993). Identification of functional angiotensin II receptors on rat cardiac fibroblasts. *Circulation*.

[46] Crawford D. C., Chobanian A. V., Brecher P. (1994). Angiotensin II induces fibronectin expression associated with cardiac fibrosis in the rat. *Circulation Research*.

[47] Ostrom R. S., Naugle J. E., Hase M. (2003). Angiotensin II enhances adenylyl cyclase signaling via Ca^2+^/calmodulin. G_q_-G_s_ cross-talk regulates collagen production in cardiac fibroblasts. *The Journal of Biological Chemistry*.

[48] Zhou G., Kandala J. C., Tyagi S. C., Katwa L. C., Weber K. T. (1996). Effects of angiotensin II and aldosterone on collagen gene expression and protein turnover in cardiac fibroblasts. *Molecular and Cellular Biochemistry*.

[49] Zhang H., Schmeißer A., Garlichs C. D. (1999). Angiotensin II-induced superoxide anion generation in human vascular endothelial cells: role of membrane-bound NADH-/NADPH-oxidases. *Cardiovascular Research*.

[50] Pastore L., Tessitore A., Martinotti S. (1999). Angiotensin II stimulates intercellular adhesion molecule-1 (ICAM-1) expression by human vascular endothelial cells and increases soluble ICAM-1 release in vivo. *Circulation*.

[51] Thomas M. C., Pickering R. J., Tsorotes D. (2010). Genetic Ace2 deficiency accentuates vascular inflammation and atherosclerosis in the ApoE knockout mouse. *Circulation Research*.

[52] Rajagopalan S., Kurz S., Münzel T. (1996). Angiotensin II-mediated hypertension in the rat increases vascular superoxide production via membrane NADH/NADPH oxidase activation: contribution to alterations of vasomotor tone. *Journal of Clinical Investigation*.

[53] Geisterfer A. A., Peach M. J., Owens G. K. (1988). Angiotensin II induces hypertrophy, not hyperplasia, of cultured rat aortic smooth muscle cells. *Circulation Research*.

[54] Itoh H., Mukoyama M., Pratt R. E., Gibbons G. H., Dzau V. J. (1993). Multiple autocrine growth factors modulate vascular smooth muscle cell growth response to angiotensin II. *Journal of Clinical Investigation*.

[55] Daemen M. J. A. P., Lombardi D. M., Bosman F. T., Schwartz S. M. (1991). Angiotensin II induces smooth muscle cell proliferation in the normal and injured rat arterial wall. *Circulation Research*.

[56] Bell L., Madri J. A. (1990). Influence of the angiotensin system on endothelial and smooth muscle cell migration. *American Journal of Pathology*.

[57] Dubey R. K., Jackson E. K., Lüscher T. F. (1995). Nitric oxide inhibits angiotensin II-induced migration of rat aortic smooth muscle cell. Role of cyclic-nucleotides and angiotensin1 receptors. *The Journal of Clinical Investigation*.

[58] Scott-Burden T., Hahn A. W. A., Resink T. J., Buhler F. R. (1990). Modulation of extracellular matrix by angiotensin II: stimulated glycoconjugate synthesis and growth in vascular smooth muscle cells. *Journal of Cardiovascular Pharmacology*.

[59] Hahn A. W. A., Jonas U., Bühler F. R., Resink T. J. (1994). Activation of human peripheral monocytes by angiotensin II. *FEBS Letters*.

[60] Lijnen P., Petrov V. (1999). Renin-angiotensin system, hypertrophy and gene expression in cardiac myocytes. *Journal of Molecular and Cellular Cardiology*.

[61] Schnee J. M., Hsueh W. A. (2000). Angiotensin II, adhesion, and cardiac fibrosis. *Cardiovascular Research*.

[62] Fabris B., Candido R., Bortoletto M. (2007). Dose and time-dependent apoptotic effects by angiotensin II infusion on left ventricular cardiomyocytes. *Journal of Hypertension*.

[63] Fabris B., Candido R., Bortoletto M. (2011). Stimulation of cardiac apoptosis in ovariectomized hypertensive rats: potential role of the renin-angiotensin system. *Journal of Hypertension*.

[64] Campbell D. J. (1987). Circulating and tissue angiotensin systems. *Journal of Clinical Investigation*.

[65] Dzau V. J. (1988). Circulating versus local renin-angiotensin system in cardiovascular homeostasis. *Circulation*.

[66] Johnston C. I. (1992). Renin-angiotensin system: a dual tissue and hormonal system for cardiovascular control. *Journal of Hypertension*.

[67] Dzau V. J., Re R. (1994). Tissue angiotensin system in cardiovascular medicine: a paradigm shift?. *Circulation*.

[68] Paul M., Wagner J., Dzau V. J. (1993). Gene expression of the renin-angiotensin system in human tissues: quantitative analysis by the polymerase chain reaction. *Journal of Clinical Investigation*.

[69] Mendelsohn F. A. O. (1985). Localization and properties of angiotensin receptors. *Journal of Hypertension*.

[70] Inagami T., Iwai 1 N., Sasaki 1 K. (1992). Cloning, expression and regulation of angiotensin II receptors. *Journal of Hypertension*.

[71] Johnston C. I. (1994). Tissue angiotensin converting enzyme in cardiac and vascular hypertrophy, repair, and remodeling. *Hypertension*.

[72] Kumar R., Yong Q. C., Thomas C. M., Baker K. M. (2012). Intracardiac intracellular angiotensin system in diabetes. *American Journal of Physiology—Regulatory Integrative and Comparative Physiology*.

[73] Bernardi S., Toffoli B., Zennaro C. (2012). High-salt diet increases glomerular ACE/ACE2 ratio leading to oxidative stress and kidney damage. *Nephrology, Dialysis, Transplantation*.

[74] Schunkert H., Dzau V. J., Tang S. S., Hirsch A. T., Apstein C. S., Lorell B. H. (1990). Increased rat cardiac angiotensin converting enzyme activity and mRNA expression in pressure overload left ventricular hypertrophy: effects on coronary resistance, contractility, and relaxation. *Journal of Clinical Investigation*.

[75] Hokimoto S., Yasue H., Fujimoto K. (1996). Expression of angiotensin-converting enzyme in remaining viable myocytes of human ventricles after myocardial infarction. *Circulation*.

[76] Hirsch A. T., Talsness C. E., Schunkert H., Paul M., Dzau V. J. (1991). Tissue-specific activation of cardiac angiotensin converting enzyme in experimental heart failure. *Circulation Research*.

[77] Pieruzzi F., Abassi Z. A., Keiser H. R. (1995). Expression of renin-angiotensin system components in the heart, kidneys, and lungs of rats with experimental heart failure. *Circulation*.

[78] Wollert K. C., Drexler H. (1999). The renin-angiotensin system and experimental heart failure. *Cardiovascular Research*.

[79] Hirsch A. T., Pinto Y. M., Schunkert H., Dzau V. J. (1990). Potential role of the tissue renin-angiotensin system in the pathophysiology of congestive heart failure. *The American Journal of Cardiology*.

[80] Lindholm L. H., Ibsen H., Dahlöf B. (2002). Cardiovascular morbidity and mortality in patients with diabetes in the Losartan intervention for endpoint reduction in hypertension study (LIFE): a randomised trial against atenolol. *The Lancet*.

[81] Daly C. A., Fox K. M., Remme W. J., Bertrand M. E., Ferrari R., Simoons M. L. (2005). The effect of perindopril on cardiovascular morbidity and mortality in patients with diabetes in the EUROPA study: results from the PERSUADE substudy. *European Heart Journal*.

[82] Patel A., Group A. C., MacMahon S. (2007). Effects of a fixed combination of perindopril and indapamide on macrovascular and microvascular outcomes in patients with type 2 diabetes mellitus (the ADVANCE trial): a randomised controlled trial. *The Lancet*.

[83] Yusuf S., Teo K. K., Pogue J. (2008). Telmisartan, ramipril, or both in patients at high risk for vascular events. *The New England Journal of Medicine*.

[84] Pitt B., Zannad F., Remme W. J. (1999). The effect of spironolactone on morbidity and mortality in patients with severe heart failure. Randomized Aldactone Evaluation Study Investigators. *The New England Journal of Medicine*.

[85] Pitt B., Remme W., Zannad F. (2003). Eplerenone, a selective aldosterone blocker, in patients with left ventricular dysfunction after myocardial infarction. *The New England Journal of Medicine*.

[86] Yamamuro M., Yoshimura M., Nakayama M. (2006). Direct effects of aldosterone on cardiomyocytes in the presence of normal and elevated extracellular sodium. *Endocrinology*.

[87] Hayashi H., Kobara M., Abe M. (2008). Aldosterone nongenomically produces NADPH oxidase-dependent reactive oxygen species and induces myocyte apoptosis. *Hypertension Research*.

[88] Stockand J. D., Meszaros J. G. (2003). Aldosterone stimulates proliferation of cardiac fibroblasts by activating Ki-RasA and MAPK1/2 signaling. *American Journal of Physiology-Heart and Circulatory Physiology*.

[89] Brilla C. G., Zhou G., Matsubara L., Weber K. T. (1994). Collagen metabolism in cultured adult rat cardiac fibroblasts: response to angiotensin II and aldosterone. *Journal of Molecular and Cellular Cardiology*.

[90] Bunda S., Wang Y., Mitts T. F. (2009). Aldosterone stimulates elastogenesis in cardiac fibroblasts via mineralocorticoid receptor-independent action involving the consecutive activation of G*α*13, c-Src, the insulin-like growth factor-I receptor, and phosphatidylinositol 3-kinase/Akt. *Journal of Biological Chemistry*.

[91] Leopold J. A., Dam A., Maron B. A. (2007). Aldosterone impairs vascular reactivity by decreasing glucose-6-phosphate dehydrogenase activity. *Nature Medicine*.

[92] Caprio M., Newfell B. G., La Sala A. (2008). Functional mineralocorticoid receptors in human vascular endothelial cells regulate intercellular adhesion molecule-1 expression and promote leukocyte adhesion. *Circulation Research*.

[93] Min L.-J., Mogi M., Li J.-M., Iwanami J., Iwai M., Horiuchi M. (2005). Aldosterone and angiotensin II synergistically induce mitogenic response in vascular smooth muscle cells. *Circulation Research*.

[94] Montezano A. C., Callera G. E., Yogi A. (2008). Aldosterone and angiotensin II synergistically stimulate migration in vascular smooth muscle cells through c-Src-regulated Redox-sensitive RhoA pathways. *Arteriosclerosis, Thrombosis, and Vascular Biology*.

[95] Pruthi D., Mccurley A., Aronovitz M., Galayda C., Karumanchi S. A., Jaffe I. Z. (2014). Aldosterone promotes vascular remodeling by direct effects on smooth muscle cell mineralocorticoid receptors. *Arteriosclerosis, Thrombosis, and Vascular Biology*.

[96] Grossmann C., Krug A. W., Freudinger R., Mildenberger S., Voelker K., Gekle M. (2007). Aldosterone-induced EGFR expression: interaction between the human mineralocorticoid receptor and the human EGFR promoter. *American Journal of Physiology—Endocrinology and Metabolism*.

[97] Usher M. G., Duan S. Z., Ivaschenko C. Y. (2010). Myeloid mineralocorticoid receptor controls macrophage polarization and cardiovascular hypertrophy and remodeling in mice. *Journal of Clinical Investigation*.

[98] Keidar S., Kaplan M., Pavlotzky E. (2004). Aldosterone administration to mice stimulates macrophage NADPH oxidase and increases atherosclerosis development: a possible role for angiotensin-converting enzyme and the receptors for angiotensin ii and aldosterone. *Circulation*.

[99] Qin W., Rudolph A. E., Bond B. R. (2003). Transgenic model of aldosterone-driven cardiac hypertrophy and heart failure. *Circulation Research*.

[100] Young M., Fullerton M., Dilley R., Funder J. (1994). Mineralocorticoids, hypertension, and cardiac fibrosis. *The Journal of Clinical Investigation*.

[101] Reil J.-C., Hohl M., Selejan S. (2012). Aldosterone promotes atrial fibrillation. *European Heart Journal*.

[102] Sun Y., Zhang J., Lu L., Chen S. S., Quinn M. T., Weber K. T. (2002). Aldosterone-induced inflammation in the rat heart: role of oxidative stress. *American Journal of Pathology*.

[103] Rajagopalan S., Duquaine D., King S., Pitt B., Patel P. (2002). Mineralocorticoid receptor antagonism in experimental atherosclerosis. *Circulation*.

[104] O'Keefe J. H., Abuissa H., Pitt B. (2008). Eplerenone improves prognosis in postmyocardial infarction diabetic patients with heart failure: results from EPHESUS. *Diabetes, Obesity and Metabolism*.

[105] Pitt B., Anker S. D., Böhm M. (2015). Rationale and design of MinerAlocorticoid Receptor antagonist Tolerability Study-Heart Failure (ARTS-HF): a randomized study of finerenone vs. eplerenone in patients who have worsening chronic heart failure with diabetes and/or chronic kidney disease. *European Journal of Heart Failure*.

[106] Donoghue M., Hsieh F., Baronas E. (2000). A novel angiotensin-converting enzyme-related carboxypeptidase (ACE2) converts angiotensin I to angiotensin 1–9. *Circulation Research*.

[107] Tipnis S. R., Hooper N. M., Hyde R., Karran E., Christie G., Turner A. J. (2000). A human homolog of angiotensin-converting enzyme. Cloning and functional expression as a captopril-insensitive carboxypeptidase. *The Journal of Biological Chemistry*.

[108] Vickers C., Hales P., Kaushik V. (2002). Hydrolysis of biological peptides by human angiotensin-converting enzyme-related carboxypeptidase. *The Journal of Biological Chemistry*.

[109] Rice G. I., Thomas D. A., Grant P. J., Turner A. J., Hooper N. M. (2004). Evaluation of angiotensin-converting enzyme (ACE), its homologue ACE2 and neprilysin in angiotensin peptide metabolism. *Biochemical Journal*.

[110] Ferrario C. M., Chappell M. C., Tallant E. A., Brosnihan K. B., Diz D. I. (1997). Counterregulatory actions of angiotensin-(1–7). *Hypertension*.

[111] Santos R. A. S., Simoes e Silva A. C., Maric C. (2003). Angiotensin-(1–7) is an endogenous ligand for the G protein-coupled receptor Mas. *Proceedings of the National Academy of Sciences of the United States of America*.

[112] Tallant E. A., Ferrario C. M., Gallagher P. E. (2005). Angiotensin-(1-7) inhibits growth of cardiac myocytes through activation of the mas receptor. *American Journal of Physiology—Heart and Circulatory Physiology*.

[113] Flores-Muñoz M., Godinho B. M. D. C., Almalik A., Nicklin S. A. (2012). Adenoviral delivery of angiotensin-(1–7) or angiotensin-(1–9) inhibits cardiomyocyte hypertrophy via the mas or angiotensin type 2 receptor. *PLoS ONE*.

[114] Niu X., Xue Y., Li X. (2014). Effects of angiotensin-(1-7) on the proliferation and collagen synthesis of arginine vasopressin–stimulated rat cardiac fibroblasts: role of mas receptor-calcineurin-NF-*κ*B signaling pathway. *Journal of Cardiovascular Pharmacology*.

[115] McCollum L. T., Gallagher P. E., Tallant E. A. (2012). Angiotensin-(1–7) abrogates mitogen-stimulated proliferation of cardiac fibroblasts. *Peptides*.

[116] Sampaio W. O., Dos Santos R. A. S., Faria-Silva R., Da Mata Machado L. T., Schiffrin E. L., Touyz R. M. (2007). Angiotensin-(1–7) through receptor Mas mediates endothelial nitric oxide synthase activation via Akt-dependent pathways. *Hypertension*.

[117] Zhang Y.-H., Zhang Y.-H., Dong X.-F. (2015). ACE2 and Ang-(1–7) protect endothelial cell function and prevent early atherosclerosis by inhibiting inflammatory response. *Inflammation Research*.

[118] Freeman E. J., Chisolm G. M., Ferrario C. M., Tallant E. A. (1996). Angiotensin-(1–7) inhibits vascular smooth muscle cell growth. *Hypertension*.

[119] Tallant E. A., Diz D. I., Ferrario C. M. (1999). Antiproliferative actions of angiotensin-(1–7) in vascular smooth muscle. *Hypertension*.

[120] Souza L. L., Costa-Neto C. M. (2012). Angiotensin-(1–7) decreases LPS-induced inflammatory response in macrophages. *Journal of Cellular Physiology*.

[121] Ferreira A. J., Santos R. A., Almeida A. P. (2001). Angiotensin-(1-7): cardioprotective effect in myocardial ischemia/reperfusion. *Hypertension*.

[122] Grobe J. L., Mecca A. P., Mao H., Katovich M. J. (2006). Chronic angiotensin-(1–7) prevents cardiac fibrosis in DOCA-salt model of hypertension. *American Journal of Physiology-Heart and Circulatory Physiology*.

[123] Ferreira A. J., Oliveira T. L., Castro M. C. M. (2007). Isoproterenol-induced impairment of heart function and remodeling are attenuated by the nonpeptide angiotensin-(1–7) analogue AVE 0991. *Life Sciences*.

[124] Santiago N. M., Guimarães P. S., Sirvente R. A. (2010). Lifetime overproduction of circulating angiotensin-(1–7) attenuates deoxycorticosterone acetate-salt hypertension-induced cardiac dysfunction and remodeling. *Hypertension*.

[125] Gomes E. R. M., Lara A. A., Almeida P. W. M. (2010). Angiotensin-(1-7) prevents cardiomyocyte pathological remodeling through a nitric oxide/guanosine 3′,5′-cyclic monophosphate-dependent pathway. *Hypertension*.

[126] Santos R. A. S., Castro C. H., Gava E. (2006). Impairment of in vitro and in vivo heart function in angiotensin-(1-7) receptor mas knockout mice. *Hypertension*.

[127] Brosnihan K. B., Li P., Ferrario C. M. (1996). Angiotensin-(1–7) dilates canine coronary arteries through kinins and nitric oxide. *Hypertension*.

[128] Tesanovic S., Vinh A., Gaspari T. A., Casley D., Widdop R. E. (2010). Vasoprotective and atheroprotective effects of angiotensin (1–7) in apolipoprotein E-deficient mice. *Arteriosclerosis, Thrombosis, and Vascular Biology*.

[129] Bosnyak S., Jones E. S., Christopoulos A., Aguilar M.-I., Thomas W. G., Widdop R. E. (2011). Relative affinity of angiotensin peptides and novel ligands at AT1 and AT2 receptors. *Clinical Science*.

[130] Raizada M. K., Ferreira A. J. (2007). ACE2: a new target for cardiovascular disease therapeutics. *Journal of Cardiovascular Pharmacology*.

[131] Patel V. B., Zhong J. C., Grant M. B., Oudit G. Y. (2016). Role of the ACE2/angiotensin 1–7 axis of the renin-angiotensin system in heart failure. *Circulation Research*.

[132] Bernardi S., Burns W. C., Toffoli B. (2012). Angiotensin-converting enzyme 2 regulates renal atrial natriuretic peptide through angiotensin-(1–7). *Clinical Science*.

[133] Nguyen G., Delarue F., Burcklé C., Bouzhir L., Giller T., Sraer J.-D. (2002). Pivotal role of the renin/prorenin receptor in angiotensin II production and cellular responses to renin. *The Journal of Clinical Investigation*.

[134] Nguyen G., Muller D. N. (2010). The biology of the (pro)renin receptor. *Journal of the American Society of Nephrology*.

[135] Hirose T., Hashimoto M., Totsune K. (2009). Association of (pro)renin receptor gene polymorphism with blood pressure in Japanese men: the Ohasama study. *American Journal of Hypertension*.

[136] Saris J. J., 't Hoen P. A. C., Garrelds I. M. (2006). Prorenin induces intracellular signaling in cardiomyocytes independently of angiotensin II. *Hypertension*.

[137] Véniant M., Ménard J., Bruneval P., Morley S., Gonzales M. F., Mullins J. (1996). Vascular damage without hypertension in transgenic rats expressing prorenin exclusively in the liver. *The Journal of Clinical Investigation*.

[138] Saris J. J., van den Eijnden M. M. E. D., Lamers J. M. J., Saxena P. R., Schalekamp M. A. D. H., Danser A. H. J. (2002). Prorenin-induced myocyte proliferation: no role for intracellular angiotensin II. *Hypertension*.

[139] Hinrichs S., Heger J., Schreckenberg R. (2011). Controlling cardiomyocyte length: the role of renin and PPAR-*γ*. *Cardiovascular Research*.

[140] Uraoka M., Ikeda K., Nakagawa Y. (2009). Prorenin induces ERK activation in endothelial cells to enhance neovascularization independently of the renin-angiotensin system. *Biochemical and Biophysical Research Communications*.

[141] Sakoda M., Ichihara A., Kaneshiro Y. (2007). (Pro)renin receptor-mediated activation of mitogen-activated protein kinases in human vascular smooth muscle cells. *Hypertension Research*.

[142] Liu F. Y., Liu X. Y., Zhang L. J., Cheng Y. P., Jiang Y. N. (2014). Binding of prorenin to (pro)renin receptor induces the proliferation of human umbilical artery smooth muscle cells via ROS generation and ERK1/2 activation. *JRAAS-Journal of the Renin-Angiotensin-Aldosterone System*.

[143] Narumi K., Hirose T., Sato E. (2015). A functional (pro)renin receptor is expressed in human lymphocytes and monocytes. *American Journal of Physiology—Renal Physiology*.

[144] Moilanen A.-M., Rysä J., Serpi R. (2012). (Pro)renin receptor triggers distinct angiotensin ii-independent extracellular matrix remodeling and deterioration of cardiac function. *PLoS ONE*.

[145] Kanda A., Noda K., Saito W., Ishida S. (2012). (Pro)renin receptor is associated with angiogenic activity in proliferative diabetic retinopathy. *Diabetologia*.

[146] Peters B., Grisk O., Becher B. (2008). Dose-dependent titration of prorenin and blood pressure in Cyp1a1ren-2 transgenic rats: absence of prorenin-induced glomerulosclerosis. *Journal of Hypertension*.

[147] Mercure C., Prescott G., Lacombe M.-J., Silversides D. W., Reudelhuber T. L. (2009). Chronic increases in circulating prorenin are not associated with renal or cardiac pathologies. *Hypertension*.

[148] Batenburg W. W., Jan Danser A. H. (2012). (Pro)renin and its receptors: pathophysiological implications. *Clinical Science*.

[149] Nishi T., Forgac M. (2002). The vacuolar (H^+^)-ATPases—nature's most versatile proton pumps. *Nature Reviews Molecular Cell Biology*.

[150] Advani A., Kelly D. J., Cox A. J. (2009). The (Pro)renin receptor: site-specific and functional linkage to the vacuolar H^+^-atpase in the kidney. *Hypertension*.

[151] Ramser J., Abidi F. E., Burckle C. A. (2005). A unique exonic splice enhancer mutation in a family with X-linked mental retardation and epilepsy points to a novel role of the renin receptor. *Human Molecular Genetics*.

[152] Contrepas A., Walker J., Koulakoff A. (2009). A role of the (pro)renin receptor in neuronal cell differentiation. *American Journal of Physiology—Regulatory Integrative and Comparative Physiology*.

[153] Cruciat C.-M., Ohkawara B., Acebron S. P. (2010). Requirement of prorenin receptor and vacuolar H^+^-ATPase-mediated acidification for Wnt signaling. *Science*.

[154] Amsterdam A., Nissen R. M., Sun Z., Swindell E. C., Farrington S., Hopkins N. (2004). Identification of 315 genes essential for early zebrafish development. *Proceedings of the National Academy of Sciences of the United States of America*.

[155] Kinouchi K., Ichihara A., Sano M. (2010). The (pro)renin receptor/ATP6AP2 is essential for vacuolar H^+^-ATPase assembly in murine cardiomyocytes. *Circulation Research*.

[156] Rubler S., Dlugash J., Yuceoglu Y. Z., Kumral T., Branwood A. W., Grishman A. (1972). New type of cardiomyopathy associated with diabetic glomerulosclerosis. *The American Journal of Cardiology*.

[157] Miller J. A., Floras J. S., Zinman B., Skorecki K. L., Logan A. G. (1996). Effect of hyperglycaemia on arterial pressure, plasma renin activity and renal function in early diabetes. *Clinical Science*.

[158] Miller J. A. (1999). Impact of hyperglycemia on the renin angiotensin system in early human type 1 diabetes mellitus. *Journal of the American Society of Nephrology*.

[159] Osei S. Y., Price D. A., Laffel L. M. B., Lansang M. C., Hollenberg N. K. (2000). Effect of angiotensin II antagonist eprosartan on hyperglycemia-induced activation of intrarenal renin-angiotensin system in healthy humans. *Hypertension*.

[160] Solomon S. D., Appelbaum E., Manning W. J. (2009). Effect of the direct renin inhibitor aliskiren, the angiotensin receptor blocker losartan, or both on left ventricular mass in patients with hypertension and left ventricular hypertrophy. *Circulation*.

[161] Shah A. M., Shin S. H., Takeuchi M. (2012). Left ventricular systolic and diastolic function, remodelling, and clinical outcomes among patients with diabetes following myocardial infarction and the influence of direct renin inhibition with aliskiren. *European Journal of Heart Failure*.

[162] Fiordaliso F., Li B., Latini R. (2000). Myocyte death in streptozotocin-induced diabetes in rats is angiotensin II- dependent. *Laboratory Investigation*.

[163] Singh V. P., Le B., Bhat V. B., Baker K. M., Kumar R. (2007). High-glucose-induced regulation of intracellular ANG II synthesis and nuclear redistribution in cardiac myocytes. *American Journal of Physiology-Heart and Circulatory Physiology*.

[164] Singh V. P., Baker K. M., Kumar R. (2008). Activation of the intracellular renin-angiotensin system in cardiac fibroblasts by high glucose: role in extracellular matrix production. *American Journal of Physiology—Heart and Circulatory Physiology*.

[165] Tang R., Li Q., Lv L. (2010). Angiotensin II mediates the high-glucose-induced endothelial-to-mesenchymal transition in human aortic endothelial cells. *Cardiovascular Diabetology*.

[166] Frustaci A., Kajstura J., Chimenti C. (2000). Myocardial cell death in human diabetes. *Circulation Research*.

[167] Fiordaliso F., Leri A., Cesselli D. (2001). Hyperglycemia activates p53 and p53-regulated genes leading to myocyte cell death. *Diabetes*.

[168] Singh V. P., Le B., Khode R., Baker K. M., Kumar R. (2008). Intracellular angiotensin II production in diabetic rats is correlated with cardiomyocyte apoptosis, oxidative stress, and cardiac fibrosis. *Diabetes*.

[169] Natarajan R., Scott S., Wei B., Yerneni K. K. V., Nadler J. (1999). Angiotensin II signaling in vascular smooth muscle cells under high glucose conditions. *Hypertension*.

[170] Arun K. H. S., Kaul C. L., Ramarao P. (2004). High glucose concentration augments angiotensin II mediated contraction via AT1 receptors in rat thoracic aorta. *Pharmacological Research*.

[171] Sechi L. A., Griffin C. A., Schambelan M. (1994). The cardiac renin-angiotensin system in STZ-induced diabetes. *Diabetes*.

[172] Tikellis C., Pickering R., Tsorotes D. (2012). Interaction of diabetes and ACE2 in the pathogenesis of cardiovascular disease in experimental diabetes. *Clinical Science*.

[173] Xue C., Siragy H. M. (2005). Local renal aldosterone system and its regulation by salt, diabetes, and angiotensin II type 1 receptor. *Hypertension*.

[174] Lee S. H., Yoo T.-H., Nam B.-Y. (2009). Activation of local aldosterone system within podocytes is involved in apoptosis under diabetic conditions. *American Journal of Physiology—Renal Physiology*.

[175] Fujisaki M., Nagoshi T., Nishikawa T., Date T., Yoshimura M. (2013). Rapid induction of aldosterone synthesis in cultured neonatal rat cardiomyocytes under high glucose conditions. *BioMed Research International*.

[176] An D. Z., Cat A. N. D., Soukaseum C. (2008). Cross-talk between mineralocorticoid and angiotensin II signaling for cardiac remodeling. *Hypertension*.

[177] Lemarié C. A., Paradis P., Schiffrin E. L. (2008). New insights on signaling cascades induced by cross-talk between angiotensin II and aldosterone. *Journal of Molecular Medicine*.

[178] Bernardi S., Toffoli B., Zennaro C. (2015). Aldosterone effects on glomerular structure and function. *Journal of the Renin-Angiotensin-Aldosterone System*.

[179] Yamamuro M., Yoshimura M., Nakayama M. (2008). Aldosterone, but not angiotensin II, reduces angiotensin converting enzyme 2 gene expression levels in cultured neonatal rat cardiomyocytes. *Circulation Journal*.

[180] Mandavia C. H., Aroor A. R., Demarco V. G., Sowers J. R. (2013). Molecular and metabolic mechanisms of cardiac dysfunction in diabetes. *Life Sciences*.

[181] Thomas M. C., Tikellis C., Burns W. M. (2005). Interactions between renin angiotensin system and advanced glycation in the kidney. *Journal of the American Society of Nephrology*.

[182] Nickenig G., Jung O., Strehlow K. (1997). Hypercholesterolemia is associated with enhanced angiotensin AT1-receptor expression. *American Journal of Physiology—Heart and Circulatory Physiology*.

[183] Nickenig G., Bäumer A. T., Temur Y., Kebben D., Jockenhövel F., Böhm M. (1999). Statin-sensitive dysregulated AT1 receptor function and density in hypercholesterolemic men. *Circulation*.

[184] Gurantz D., Cowling R. T., Villarreal F. J., Greenberg B. H. (1999). Tumor necrosis factor-*α* upregulates angiotensin II type 1 receptors on cardiac fibroblasts. *Circulation Research*.

[185] Urata H., Healy B., Stewart R. W., Bumpus F. M., Husain A. (1990). Angiotensin II-forming pathways in normal and failing human hearts. *Circulation Research*.

[186] Urata H., Kinoshita A., Perez D. M. (1991). Cloning of the gene and cDNA for human heart chymase. *The Journal of Biological Chemistry*.

[187] Huynh K., Bernardo B. C., McMullen J. R., Ritchie R. H. (2014). Diabetic cardiomyopathy: mechanisms and new treatment strategies targeting antioxidant signaling pathways. *Pharmacology and Therapeutics*.

[188] Tikellis C., Bialkowski K., Pete J. (2008). ACE2 deficiency modifies renoprotection afforded by ACE inhibition in experimental diabetes. *Diabetes*.

[189] Tikellis C., Bernardi S., Burns W. C. (2011). Angiotensin-converting enzyme 2 is a key modulator of the renin-angiotensin system in cardiovascular and renal disease. *Current Opinion in Nephrology and Hypertension*.

[190] Wei C.-C., Tian B., Perry G. (2002). Differential ANG II generation in plasma and tissue of mice with decreased expression of the ACE gene. *American Journal of Physiology—Heart and Circulatory Physiology*.

[191] Li C., Cao L., Zeng Q. (2005). Taurine may prevent diabetic rats from developing cardiomyopathy also by downregulating angiotensin II type2 receptor expression. *Cardiovascular Drugs and Therapy*.

[192] Hakam A. C., Hussain T. (2005). Renal angiotensin II type-2 receptors are upregulated and mediate the candesartan-induced natriuresis/diuresis in obese Zucker rats. *Hypertension*.

[193] Mezzano S., Droguett A., Eugenia Burgos M. (2003). Renin-angiotensin system activation and interstitial inflammation in human diabetic nephropathy. *Kidney International, Supplement*.

[194] Dhande I., Ali Q., Hussain T. (2013). Proximal tubule angiotensin AT2 receptors mediate an anti-inflammatory response via interleukin-10: role in renoprotection in obese rats. *Hypertension*.

[195] Ali Q., Sabuhi R., Hussain T. (2010). High glucose up-regulates angiotensin II subtype 2 receptors via interferon regulatory factor-1 in proximal tubule epithelial cells. *Molecular and Cellular Biochemistry*.

[196] Ichihara A., Itoh H., Inagami T. (2008). Critical roles of (pro)renin receptor-bound prorenin in diabetes and hypertension: sallies into therapeutic approach. *Journal of the American Society of Hypertension*.

[197] Wilson D. M., Luetscher J. A. (1990). Plasma prorenin activity and complications in children with insulin-dependent diabetes mellitus. *New England Journal of Medicine*.

[198] Deinum J., Rønn B., Mathiesen E., Derkx F. H. M., Hop W. C. J., Schalekamp M. A. D. H. (1999). Increase in serum prorenin precedes onset of microalbuminuria in patients with insulin-dependent diabetes mellitus. *Diabetologia*.

[199] Connelly K. A., Advani A., Kim S. (2011). The cardiac (pro)renin receptor is primarily expressed in myocyte transverse tubules and is increased in experimental diabetic cardiomyopathy. *Journal of Hypertension*.

[200] McMurray J. J. V. (2015). Neprilysin inhibition to treat heart failure: a tale of science, serendipity, and second chances. *European Journal of Heart Failure*.

[201] Rademaker M. T., Charles C. J., Espiner E. A., Nicholls M. G., Richards A. M., Kosoglou T. (1998). Combined neutral endopeptidase and angiotensin-converting enzyme inhibition in heart failure: role of natriuretic peptides and angiotensin II. *Journal of Cardiovascular Pharmacology*.

[202] Trippodo N. C., Fox M., Monticello T. M., Panchal B. C., Asaad M. M. (1999). Vasopeptidase inhibition with omapatrilat improves cardiac geometry and survival in cardiomyopathic hamsters more than does ACE inhibition with captopril. *Journal of Cardiovascular Pharmacology*.

[203] Kostis J. B., Packer M., Black H. R., Schmieder R., Henry D., Levy E. (2004). Omapatrilat and enalapril in patients with hypertension: the Omapatrilat Cardiovascular Treatment vs. Enalapril (OCTAVE) trial. *American Journal of Hypertension*.

[204] Packer M., Califf R. M., Konstam M. A. (2002). Comparison of omapatrilat and enalapril in patients with chronic heart failure: the omapatrilat versus enalapril randomized trial of utility in reducing events (OVERTURE). *Circulation*.

[205] McMurray J. J. V., Packer M., Desai A. S. (2014). Angiotensin-neprilysin inhibition versus enalapril in heart failure. *The New England Journal of Medicine*.

[206] Kristensen S. L., Preiss D., Jhund P. S. (2016). Risk related to pre-diabetes mellitus and diabetes mellitus in heart failure with reduced ejection fraction: insights from prospective comparison of ARNI with ACEI to Determine Impact on Global Mortality and Morbidity in Heart Failure Trial. *Circulation: Heart Failure*.

[207] Suematsu Y., Miura S.-I., Goto M. (2016). LCZ696, an angiotensin receptor-neprilysin inhibitor, improves cardiac function with the attenuation of fibrosis in heart failure with reduced ejection fraction in streptozotocin-induced diabetic mice. *European Journal of Heart Failure*.

[208] Namsolleck P., Unger T. (2014). Aldosterone synthase inhibitors in cardiovascular and renal diseases. *Nephrology Dialysis Transplantation*.

[209] Fiebeler A., Nussberger J., Shagdarsuren E. (2005). Aldosterone synthase inhibitor ameliorates angiotensin II-induced organ damage. *Circulation*.

[210] Mulder P., Mellin V., Favre J. (2008). Aldosterone synthase inhibition improves cardiovascular function and structure in rats with heart failure: a comparison with spironolactone. *European Heart Journal*.

[211] Gamliel-Lazarovich A., Gantman A., Coleman R., Jeng A. Y., Kaplan M., Keidar S. (2010). FAD286, an aldosterone synthase inhibitor, reduced atherosclerosis and inflammation in apolipoprotein E-deficient mice. *Journal of Hypertension*.

[212] Unger T., Paulis L., Sica D. A. (2011). Therapeutic perspectives in hypertension: novel means for renin-angiotensin-aldosterone system modulation and emerging device-based approaches. *European Heart Journal*.

[213] Parving H.-H., Brenner B. M., McMurray J. J. V. (2012). Cardiorenal end points in a trial of aliskiren for type 2 diabetes. *The New England Journal of Medicine*.

[214] Schefe J. H., Neumann C., Goebel M. (2008). Prorenin engages the (pro)renin receptor like renin and both ligand activities are unopposed by aliskiren. *Journal of Hypertension*.

[215] Sealey J. E., Laragh J. H. (2007). Aliskiren, the first renin inhibitor for treating hypertension: reactive renin secretion may limit its effectiveness. *American Journal of Hypertension*.

[216] Ocaranza M. P., Godoy I., Jalil J. E. (2006). Enalapril attenuates downregulation of angiotensin-converting enzyme 2 in the late phase of ventricular dysfunction in myocardial infarcted rat. *Hypertension*.

[217] Ferrario C. M., Jessup J., Chappell M. C. (2005). Effect of angiotensin-converting enzyme inhibition and angiotensin II receptor blockers on cardiac angiotensin-converting enzyme 2. *Circulation*.

[218] Crackower M. A., Sarao R., Oudit G. Y. (2002). Angiotensin-converting enzyme 2 is an essential regulator of heart function. *Nature*.

[219] Patel V. B., Bodiga S., Basu R. (2012). Loss of angiotensin-converting enzyme-2 exacerbates diabetic cardiovascular complications and leads to systolic and vascular dysfunction: a critical role of the angiotensin II/AT1 receptor axis. *Circulation Research*.

[220] Patel V. B., Mori J., McLean B. A. (2016). ACE2 deficiency worsens epicardial adipose tissue inflammation and cardiac dysfunction in response to diet-induced obesity. *Diabetes*.

[221] Dong B., Yu Q. T., Dai H. Y. (2012). Angiotensin-converting enzyme-2 overexpression improves left ventricular remodeling and function in a rat model of diabetic cardiomyopathy. *Journal of the American College of Cardiology*.

[222] Murça T. M., Moraes P. L., Capuruço C. A. B. (2012). Oral administration of an angiotensin-converting enzyme 2 activator ameliorates diabetes-induced cardiac dysfunction. *Regulatory Peptides*.

[223] Haschke M., Schuster M., Poglitsch M. (2013). Pharmacokinetics and pharmacodynamics of recombinant human angiotensin-converting enzyme 2 in healthy human subjects. *Clinical Pharmacokinetics*.

[224] Giani J. F., Muñoz M. C., Mayer M. A. (2010). Angiotensin-(1–7) improves cardiac remodeling and inhibits growthpromoting pathways in the heart of fructose-fed rats. *American Journal of Physiology—Heart and Circulatory Physiology*.

[225] Singh K., Singh T., Sharma P. L. (2011). Beneficial effects of angiotensin (1–7) in diabetic rats with cardiomyopathy. *Therapeutic Advances in Cardiovascular Disease*.

[226] Hao P.-P., Yang J.-M., Zhang M.-X. (2015). Angiotensin-(1–7) treatment mitigates right ventricular fibrosis as a distinctive feature of diabetic cardiomyopathy. *American Journal of Physiology—Heart and Circulatory Physiology*.

[227] Papinska A. M., Mordwinkin N. M., Meeks C. J., Jadhav S. S., Rodgers K. E. (2015). Angiotensin-(1–7) administration benefits cardiac, renal and progenitor cell function in *db/db* mice. *British Journal of Pharmacology*.

[228] Mori J., Patel V. B., Alrob O. A. (2014). Angiotensin 1-7 ameliorates diabetic cardiomyopathy and diastolic dysfunction in db/db mice by reducing lipotoxicity and inflammation. *Circulation: Heart Failure*.

[229] Ebermann L., Spillmann F., Sidiropoulos M. (2008). The angiotensin-(1–7) receptor agonist AVE0991 is cardioprotective in diabetic rats. *European Journal of Pharmacology*.

[230] Loot A. E., Roks A. J. M., Henning R. H. (2002). Angiotensin-(1–7) attenuates the development of heart failure after myocardial infarction in rats. *Circulation*.

[231] Benter I. F., Yousif M. H. M., Cojocel C., Al-Maghrebi M., Diz D. I. (2007). Angiotensin-(1–7) prevents diabetes-induced cardiovascular dysfunction. *American Journal of Physiology—Heart and Circulatory Physiology*.

[232] Bossi F., Bernardi S., De Nardo D. (2016). Angiotensin 1–7 significantly reduces diabetes-induced leukocyte recruitment both in vivo and in vitro. *Atherosclerosis*.

[233] Koulis C., Chow B. S. M., Mckelvey M. (2015). AT2R agonist, compound 21, is reno-protective against type 1 diabetic nephropathy. *Hypertension*.

[234] Castoldi G., di Gioia C. R. T., Bombardi C. (2014). Prevention of diabetic nephropathy by compound 21, selective agonist of angiotensin type 2 receptors, in Zucker diabetic fatty rats. *American Journal of Physiology—Renal Physiology*.

[235] Sampson A. K., Irvine J. C., Shihata W. A. (2016). Compound 21, a selective agonist of angiotensin AT2 receptors, prevents endothelial inflammation and leukocyte adhesion in vitro and in vivo. *British Journal of Pharmacology*.

[236] Chow B. S., Koulis C., Krishnaswamy P. (2016). The angiotensin II type 2 receptor agonist Compound 21 is protective in experimental diabetes-associated atherosclerosis. *Diabetologia*.

